# Shifts in the Human Gut Microbiota Structure Caused by Quadruple *Helicobacter pylori* Eradication Therapy

**DOI:** 10.3389/fmicb.2019.01902

**Published:** 2019-08-27

**Authors:** Evgenii I. Olekhnovich, Alexander I. Manolov, Andrey E. Samoilov, Nikita A. Prianichnikov, Maja V. Malakhova, Alexander V. Tyakht, Alexander V. Pavlenko, Vlad V. Babenko, Andrey K. Larin, Boris A. Kovarsky, Elizaveta V. Starikova, Oksana E. Glushchenko, Dilyara D. Safina, Maria I. Markelova, Eugenia A. Boulygina, Dilyara R. Khusnutdinova, Sergey Y. Malanin, Sayar R. Abdulkhakov, Rustam A. Abdulkhakov, Tatiana V. Grigoryeva, Elena S. Kostryukova, Vadim M. Govorun, Elena N. Ilina

**Affiliations:** ^1^Federal Research and Clinical Centre of Physical-Chemical Medicine of Federal Medical Biological Agency, Moscow, Russia; ^2^Kazan Federal University, Kazan, Russia; ^3^Kazan Institute of Biochemistry and Biophysics, FRC Kazan Scientific Center of RAS, Kazan, Russia; ^4^Kazan State Medical University, Kazan, Russia

**Keywords:** gut microbiota, antibiotic resistance, *Helicobacter pylory* eradication, metagenome-assembled genome, enterococci, horizontal gene transfer

## Abstract

The human gut microbiome plays an important role both in health and disease. Use of antibiotics can alter gut microbiota composition, which can lead to various deleterious events. Here we report a whole genome sequencing metagenomic/genomic study of the intestinal microbiota changes caused by *Helicobacter pylori* (HP) eradication therapy. Using approaches for metagenomic data analysis we revealed a statistically significant decrease in alpha-diversity and relative abundance of *Bifidobacterium adolescentis* due to HP eradication therapy, while the relative abundance of *Enterococcus faecium* increased. We have detected changes in general metagenome resistome profiles as well: after HP eradication therapy, the *ermB, CFX* group, and *tetQ* genes were overrepresented, while *tetO* and *tetW* genes were underrepresented. We have confirmed these results with genome-resolved metagenomic approaches. MAG (metagenome-assembled genomes) abundance profiles have changed dramatically after HP eradication therapy. Focusing on *ermB* gene conferring resistance to macrolides, which were included in the HP eradication therapy scheme, we have shown a connection between antibiotic resistance genes (ARGs) and some overrepresented MAGs. Moreover, some *E. faecium* strains isolated from stool samples obtained after HP eradication have manifested greater antibiotic resistance *in vitro* in comparison to other isolates, as well as the higher number of ARGs conferring resistance to macrolides and tetracyclines.

## 1. Background

Up to 50% of the human population are hosts of *Helicobacter pylori* (HP), which can provoke a wide range of gastrointestinal diseases, including chronic gastritis, stomach ulcers, duodenal ulcers, gastric cancer, and lymphoma of mucin-associated lymphoid tissue (Covacci et al., 1999; Sanders and Peura, 2002). This bacterium is also associated with iron-deficiency anemia, Werlhof disease in children (Queiroz et al., 2013), nutritional anemia due to vitamin B_12_ deficiency, thrombocytopenic purpura, and lipid, and glucose metabolism disorders (Buzás, 2014). Conventional HP eradication therapy regimens are based on antibiotics such as clarithromycin, amoxicillin, tetracycline, and metronidazole as well as proton pump inhibitors and other auxiliary drugs including bismuth salts (Malfertheiner et al., 2017) and probiotics. Antibiotic treatment can significantly alter gut microbial community composition which may lead to antibiotic-associated diarrhea (Bartlett, 2002; Tong et al., 2007). Bacteria can acquire antibiotic resistance through a number of mechanisms, either by single-nucleotide polymorphisms (SNPs) in antibiotic-target sites or by acquiring antibiotic resistance genes (ARGs), encoding efflux pumps, drug modifiers or proteins which protect antibiotic targets (Wright, 2007; Davies and Davies, 2010). ARGs can be transferred among bacteria by mobile genetic elements, bacteriophages, and as the result of natural transformation (Davies and Davies, 2010; Schjørring and Krogfelt, 2011; Smillie et al., 2011).

The term “resistome” has been coined to refer to the collection of ARGs in a particular bacteria or microbial community (Wright, 2007; Marshall and Levy, 2011). The resistome of human gut microbiota differs widely among the human populations of different countries and reflects patterns of antibiotic use (Forslund et al., 2013; Yarygin et al., 2017). Proton-pump inhibitors which are also included in many eradication therapy schemes may also lead to a significant shift in the gut microbial community due to an intrusion of species which cannot pass gastric acid barrier under normal conditions (Jackson et al., 2015; Imhann et al., 2016). Thus, HP eradication therapy can have a great impact on gut microbial community composition. These changes can drive gut disorders and cause accumulation of ARGs in the gut microbial community. Here we use whole genome shotgun sequencing (WGS) to study taxonomic and functional profile shifts of the gut microbial community in patients after HP eradication therapy. Our study focuses on shifts in gut microbiota driven by the complex mix of drugs used in pathogen eradication for patients with different pathologies of the gastrointestinal tract.

## 2. Methods

### 2.1. Sample Collections and HP Eradication Scheme

Fecal samples were acquired prior to and immediately following HP eradication therapy from the patients suffering from diseases associated with HP infection—gastroesophageal reflux disease, chronic gastritis, chronic duodenitis, and duodenal ulcers as well as five patients with an unclear diagnosis who took medications as a preventive measure against stomach cancer (see Table S1). In all cases, the HP infection was confirmed by urease testing and cytological examination of mucosal biopsies collected from the antral part of the stomach during gastroscopy study. All patients included in the study received the HP eradication treatment according to the Maastricht scheme (Malfertheiner et al., 2017), which included amoxicillin 1,000 mg, clarithromycin 500 mg, bismuth subsalicylate 240 mg, and proton pump inhibitors (esomeprazole/pantoprazole) 20 mg daily. The therapy course lasted for 14 days. Lactulose was used as a prebiotic throughout the therapy course. Two sampling points were included: before the treatment and 0 or 2 days after the treatment. A total of 80 fecal samples from 40 patients were collected (47.7 ± 13.11 years old, 18 females, and 22 males).

### 2.2. Samples Preparation and Sequencing

Stool samples were delivered to the laboratory and subsequently stored at –20°C. Before the DNA extraction procedure, the physical state of the samples was evaluated and their weight was determined. The weight of each sample exceeded 20 g, which was a sufficient quantity for the extraction of the required amount of DNA. The total DNA extraction was performed as described previously (Tyakht et al., 2013). The whole genome shotgun sequencing on ABI/SOLiD 5500W platform in the 50 base pairs in single-end mode was performed according to the manufacturer's recommendations (Life Technologies, Foster City, CA, USA) using the following kits: 5500W Conversion Primers Kit, 5500W FlowChip V2, 5500W FlowChip Prep Pack, 5500W Template Amplification Kit v2, 5500W FWD1 SP Kit; 5500W FWD2 SP Kit; 5500W FWD SR Kit; 5500W FWD Ligase Kit; 5500W Run Cycle Buffer Kit, and 5500W FWD Buffer; 5500W Buffer D.

### 2.3. Metagenomic Data Preprocessing

Metagenomic reads were filtered by quality. To minimize sequencing errors, remaining reads were processed with SAET software (Life Technologies) with parameters -qvupdate -trustprefix 25 -localrounds 3 -global rounds 2. Further quality filtration was carried out as following: all positions starting from 5′-end were removed up to the first high-quality position (QV > 30). Afterwards, the filtered reads shorter than 30 nucleotides were discarded. The resulting reads were 30–50 bp long with QV > 30. In order to remove the fragments of human DNA from the data, reads were mapped to the human genome hg38 using Bowtie (Langmead et al., 2009) with parameters -f -S -t -v 3 -k 1. Reads unmapped to the human genome were converted from the color space to base space and used in further analysis.

### 2.4. Additional Metagenomic Data

Ten metagenomic samples from four patients included in this study were additionally sequenced on Illumina HiSeq 2500 platform and used to test the MetaCherchant algorithm (Glushchenko et al., 2017; Olekhnovich et al., 2017). The choice of samples to undergo additional sequencing via Illumina platform was performed based on the presence of potential opportunistic bacterial taxa obtained by preliminary metagenomic analysis and bacterial isolating results. Methods for preprocessing these datasets can be found in the indicated articles.

### 2.5. *Enterococcus* spp. Isolation Scheme, Genomic Sequencing, and Assembly

*Enterococcus* spp. strains were isolated from Illumina-sequenced stool samples. In total, 19 strains were recovered from four patients; six genomes have been sequenced and presented previously (Prianichnikov et al., 2018). Here we present the remaining 13 genomes and combined analysis using all genomic data. Genomic assembly was performed using SPAdes (Bankevich et al., 2012) with default parameters.

### 2.6. Susceptibility Testing of *Enterococcus* spp. Strains

Susceptibility of isolated *Enterococcus* spp. strains to azithromycin, ciprofloxacin, tetracycline, penicillin, vancomycin and cefixime were tested using the disk diffusion method. Plates containing Mueller-Hinton agar were inoculated by pre-grown bacterial culture adjusted to a turbidity of a 1.0 McFarland, then disks with antibiotics were placed and tightly pressed onto the surfaces of the inoculated plates to ensure constant diffusion of antibiotic. The plates were incubated at 37°C in a thermostat for 24 h. Inhibition zone diameter was measured to within 1 mm taking into account the diameter of the disk itself. Only complete inhibition of visible growth was measured; the smallest colonies that were only detected under specific lighting conditions and that had a barely visible plume at the edge of the inhibition zone were not counted. Formation of large colonies within a clear zone of growth inhibition was considered as an evidence of the presence of extraneous microflora or multiple resistance of the population. In these cases, re-identification and re-testing for antibiotic resistance from both zones were conducted. Strains were considered sensitive or resistant according to the CLSI criteria (CLSI, 2015).

### 2.7. Bioinformatic and Statistical Analysis

Metagenomic taxonomic and functional profiling was performed using HUMAnN2 (Franzosa et al., 2018; contains MetaPhlAn2 Truong et al., 2015 which allows taxonomic profiling of metagenomes) and KEGG database (release 2018-03-26; Kanehisa et al., 2015), which was adapted for use in HUMAnN2 pipeline. The ARGs distributions were obtained in the metagenomes by mapping the reads to MEGARes reference database using Bowtie with keys -f -S -t -v 3 -k 1. The relative abundance of groups, mechanisms and classes of ARGs was extracted using ResistomeAnalyzer (Lakin et al., 2016) and calculating RPKM value. The GROOT (Graphing Resistance Out Of meTagenomes) tool (Rowe and Winn, 2018) and ARG-ANNOT database (Gupta et al., 2014) were used for additional resistome profiling. MAGs assembly was performed using the MetaWRAP pipeline [Uritskiy et al., 2018; contains MEGAHIT (Li et al., 2015) as a metagenomic assembler; BWA (Li et al., 2009); CONCOCT (Alneberg et al., 2013), MetaBat (Kang et al., 2015), and MaxBin2 (Wu et al., 2015) for metagenomic binning] with the following parameters: completeness > 70% and contamination < 10%. For processing and visualization of MAGs-related data we applied Anvi'o framework [Eren et al., 2015; contains Bowtie2 (Langmead and Salzberg, 2012), Prodigal (Hyatt et al., 2010), samtools (Li et al., 2009)]. CheckM (Parks et al., 2015) was used for the comparison of genomes and taxonomic annotation of MAGs. MetaCherchant was applied for the specific metagenomic assembly of the target ARGs context (Olekhnovich et al., 2017). Search of MAG sequences in the obtained contexts was performed by BLAST. Visualization of the resulting graphs was performed using Bandage (Wick et al., 2015). Mash (Ondov et al., 2016) was used for genomic comparison of *Enterococcus* spp. isolates and MAGs. Metagenomic abundance profiles of *Enterococcus* spp. isolates were obtained using metagenomic pipeline from Anvi'o framework. For functional profiling of isolates genomes, KEGG database and BLAST were used.

For data visualization and exploration analysis, CoDa approaches such as Aitchison distance (Aitchison, 1992; Aitchison and Pawlowsky-Glahn, 1997) and CoDa dendrogram were used. CoDa dendrogram is a dendrogram-like graph that shows: (a) the way of grouping parts of the compositional vector; (b) the explanatory role of each sub-composition generated in the partition process; (c) the decomposition of the total variance into balance components associated with each binary partition (Egozcue and Pawlowsky-Glahn, 2005; Pawlowsky-Glahn and Egozcue, 2011). Differences between community structures were evaluated using selbal (Rivera-Pinto et al., 2018). Before the analysis, Bayesian estimation of (non-zero) proportions was performed for removal of rare taxa and substitution of zeros (Egozcue et al., 2018).

To determine differences in relative abundance of KEGG metabolic pathways and antibiotic resistance genes, the GSA from piano Bioconductor package (Väremo et al., 2013) was used with the following parameters: gene set analysis using “reporter feature algorithm,” significance assessment using “gene sampling” with gene set significance threshold: adj. *p* < 0.05 (according to Dubinkina et al., 2017). Wilcoxon signed rank test (wilcox.test function for R with the paired parameter TRUE) was applied for the additional differential analysis for paired data. Similarly, the Wilcoxon signed rank test (wilcox.test with the paired parameter FALSE) was used for the additional differential analysis for unpaired data. Data visualization was performed using R (R-Core-Team, 2018).

## 3. Results

### 3.1. Cohorts of Patients and Available Data

**Figure 1:**
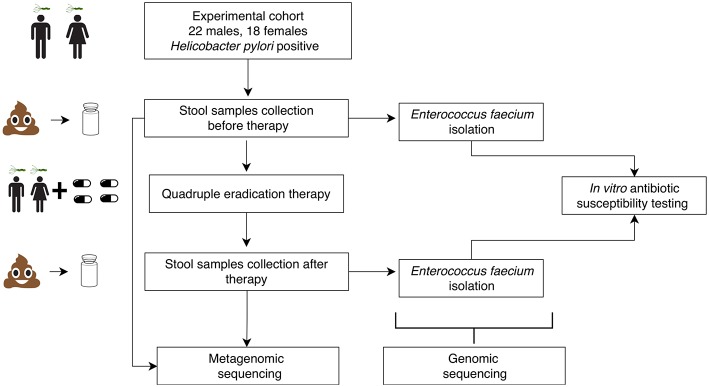
Schematic visualization of experimental design.

Eighty stool samples were collected from 40 HP-infected patients (47.7 ± 13.11 years old, 18 females and 22 males) at Kazan State University Clinical Center. The patients were diagnosed with at least one duodenal ulcer, chronic gastritis, chronic duodenitis or gastroesophageal reflux disease (GERD). Five patients had an unclear diagnosis and took medications as a preventive measure against stomach cancer. Samples were collected before and after HP eradication therapy which lasted for 14 days. The experiment scheme is presented in Figure 1. The sampling scheme is shown in Figure S1. Sequencing of the stool samples yielded 30.2 ± 10.0 M of 50 bp reads per sample (120.9 Gbp in total). Summary data about the samples and patients are presented in Table S1.

### 3.2. HP Eradication Therapy Affects Composition of Gut Microbiota

**Figure 2:**
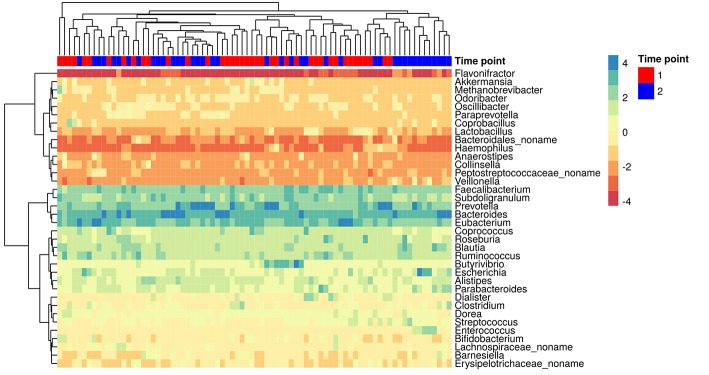
The most abundant genera in the gut microbiota of patients. Colors denote relative abundance of genera obtained by MetaPhlAn2 after clr (centered log-ratio) transformation and substitution of zeros (the higher value corresponding to the higher relative abundance). The figure shows the taxa present in at least 25% of the samples. The columns correspond to the samples/patients; the time points (1st—before HP eradication therapy, 2nd—after) are denoted with a top color bar. Hierarchical clustering was performed using the Euclidean distance and complete linkage. OTUs with no genus information are collapsed into the no-name genus.

The MetaPhlAn2 method based on unique clade-specific markers (Truong et al., 2015) allowed us to identify 105 microbial species belonging to 51 genera in the patients' metagenomes. Taxonomic relative abundances at the genera level are presented in additional materials (Table S2). Richness of the gut communities (alpha-diversity) significantly decreased after HP eradication therapy (Shannon index at genus level, Wilcoxon signed rank test, 2.45 ± 0.51 before vs. 2.17 ± 0.51 after the therapy, *p* < 0.05). Analysis of genera relative abundance demonstrated that gut communities of patients after HP eradication therapy had the tendency to reshape. *Eubacterium* (15.46 ± 13.45%), *Bacteroides* (14.52 ± 16.92%), and *Prevotella* (14.43 ± 22.37%) were the three most abundant genera before the therapy. After the eradication, the same genera were the most abundant, but their order had changed: *Bacteroides* (22.77 ± 22.06%), *Prevotella* (14.06 ± 23.34%), *Eubacterium* (10.01 ± 16.08%). The genera distribution across metagenomic samples is presented in Figure 2.

**Figure 3:**
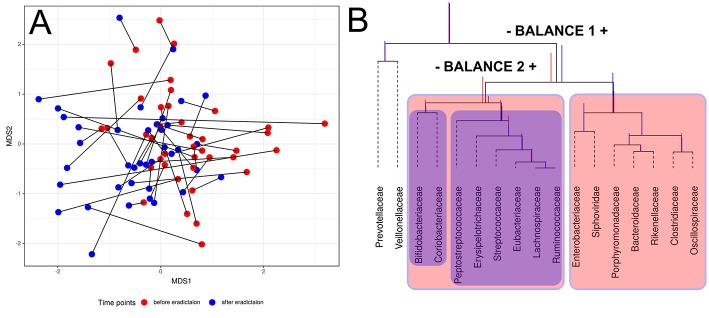
Changes of gut microbiota composition of patients. **(A)** Multidimensional scaling biplot of taxonomic profiles (genera level) of patients metagenomic samples before and after antibiotics therapy using Aitchison distance. Samples from the same patient are connected by lines. **(B)** CoDa dendrogram which characterizes the association of bacterial and bacteriophage families with balances presented as edges. Decomposition of total variance by balances between groups of families is shown using vertical bars. Mean values of balances are shown using anchoring points of vertical bars (according to Pawlowsky-Glahn and Egozcue, 2011). Red color bar denotes the 1st-time point (before HP eradication), whereas the 2nd-time point is shown using a blue color bar. The red color area indicates Balance 1, and Balance 2 is shown using the blue color area.

Compositional data (CoDa) approaches for analysis such as Aitchison distance and CoDa dendrogram were used to describe specific taxonomic patterns (see Figure 3). The taxa present in at least 25% of the samples were analyzed. Using this approach, we have shown the change of taxonomic composition between two states. Figure 3A shows the absence of clear clustering by time points; however, patterns of consistent movement can be described from right to left. CoDa dendrogram approach is based on “balances” which can be defined as log-ratios of the geometric means of the group of parts (Rivera-Pinto et al., 2018). In our case, it is relative abundance in microbial taxa. CoDa dendrogram is presented in Figure 3B.

According to the dendrogram, the left part of the Balance 1 (indicated “–Balance 1” on Figure 3B) includes eight bacterial families (*Bifidobacteriacea, Coriobacteriacea, Peptostreptococcaceae, Erysipelotrichaceae, Streptococcaceae, Eubacteriaceae, Lachnospiraceae*, and *Ruminococcaceae*) associated with the post-HP eradication time point. Moreover, *Bifidobacteriaceae* and *Coriobacteriaceae* show a stronger link with the first time point than the rest of this list. On the other hand, the right part of the Balance 1 (indicated “Balance 1 +” on Figure 3B) includes *Enterobacteriaceae, Porphyromonadaceae, Bacteroidaceae, Rikenellaceae, Clostridiaceae*, and *Oscillospiracea*, which are associated with the second time point. Interestingly, one of the bacteriophages families—*Siphoviridae*—also has a tendency to increase in relative abundance after HP eradication. This analysis indicates the association of taxonomic structure and time point (before or after treatment).

We used the selection of balances for microbial signatures (selbal) to uncover statistically significant features associated with the HP eradication therapy. Selbal implements a forward-selection method for the identification of two taxonomic groups where relative abundance, or balance, is associated with the response variable of interest (Rivera-Pinto et al., 2018). The following important features were identified based on cross-validation importance: *Bifidobacterium, Enterococcus, Flavonifractor, Coprobacillus, Collinsella*, and *Coprococcus* at genera level and 38 more features at the species level. However, a few taxonomic features were enough for significant discrimination of groups. So, according to selbal analysis, *Bifidobacterium* (*adolescentis*) significantly decreased in relative abundance after HP eradication, while *Enterococcus* (*faecium*) increased. The selbal analysis output of genera and species levels is presented in Figure S2.

### 3.3. HP Eradication Therapy Affects the Functions of Gut Microbiota

We have estimated the functional capacity of patients' gut microbiomes before and after HP eradication therapy (see Table S3). The HUMAnN2 method (Franzosa et al., 2018) allowed us to identify 4945 KEGG orthology groups (KO), which were combined into 123 KEGG pathways. Gene set analysis (GSA) revealed that seven KEGG pathways were underrepresented following HP eradication treatment. These pathways were associated with cellular processes (ko02030: Bacterial chemotaxis; ko02024: Quorum sensing; ko02040: Flagellar assembly), genetic information processing (ko00970: Aminoacyl-tRNA biosynthesis; ko03010: Ribosome) and environmental Information processing (ko02010: ABC transporters; ko02020: Two-component system). Five overrepresented KEGG pathways were associated with the metabolism of cofactors and vitamins (ko00130: Ubiquinone and other terpenoid-quinone biosynthesis; ko00785: Lipoic acid metabolism), glycan biosynthesis and metabolism (ko00511: Other glycan degradation; ko00540: Lipopolysaccharide biosynthesis). The summary statistics are presented in Table S4.

To detect more subtle functional perturbations in the gut microbiome, we have analyzed shifts in ARGs distributions. Using the MEGARes database we have detected 135 gene groups before and after HP eradication therapy conferring resistance to 16 classes of antibiotics in the metagenomes (Table S5). In general, the number of different ARGs observed increased following HP eradication therapy (Wilcoxon signed rank test *p* < 0.01), particularly, the relative abundance of 23S rRNA methyltransferases—which are associated with macrolide resistance—increased significantly (GSA, adj. *p* < 0.01). The number of SNVs in 23S rRNA methyltransferases had also increased after therapy (Wilcoxon signed rank test *p* < 0.05). The relative abundance of genes conferring tetracycline resistance (tetracycline resistance ribosomal protection proteins) had decreased (GSA, adj. *p* < 0.05). At a lower level of gene groups, *ermB* (MLS) (GSA, adj. *p* < 0.01), *CFX* group (beta-lactams), and *tetQ* (tetracyclines) genes (GSA, adj. *p* < 0.05) were increased in relative abundance; at the same time, the *tetW* and *tetO* (GSA, adj. *p* < 0.01) decreased in abundance. The results are presented in Table S6.

### 3.4. Extended Analysis of Gut Microbiota Perturbance With Genome-Resolved Metagenomics

**Figure 4:**
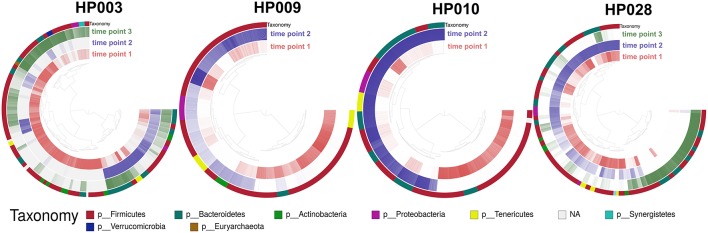
Change of the MAGs detection value depending on the time point. Each arc corresponds to one patient. Colors represent time points [red—before HP eradication therapy (1st time point), blue and green—after HP eradication therapy (2nd time points—at least 2 days and 3rd—after 2 weeks)]. Detection value (proportion of nucleotides in a contig that are covered at least 1x (according to http://merenlab.org/2017/05/08/anvio-views) was used as an abundance metric, which is shown as color brightness. Taxonomy at phylum level is shown as outer arc.

Several metagenomic samples were selected for sequencing on the Illumina HiSeq 2500 platform for a more detailed study. This data was presented earlier (Glushchenko et al., 2017) and was used for testing the MetaCherchant algorithm (Olekhnovich et al., 2017). It is worth noting that for two patients (HP003 and HP010), the third time point samples were collected two weeks after HP eradication therapy. To deepen our study from general changes in gut communities to the level of individual bacterial genomes, we used binning and assembly techniques of the metagenome-assembled genomes (MAGs). MAGs were assembled using the metaWRAP pipeline (Uritskiy et al., 2018); for each individual sample set, 53 ± 31 (215 in a total) MAGs were identified. Using the Anvi'o framework, the reads coverage profiles were obtained for each MAG at each time point. Detection value (proportions of nucleotides in a contig that are covered at least 1x; Eren et al., 2015) was used as an abundance metric (see Figure 4).

Distribution of MAGs changed dramatically after HP eradication therapy (107 MAGs were underrepresented, 71 MAGs were overrepresented, 37 were at the same level as prior to therapy). Interestingly, MAG abundance changed individually for each patient, but some general patterns can be distinguished. For example, after HP eradication therapy, for the majority of *Actinobacteria* MAGs (unknown *Coriobacteriaceae*, unknown *Bifidobacterium*, and *Bifidobacterium adolescentis*) and other bacterial taxa including *Synergistia* and *Verrucomicrobiae* detection value had decreased, and the detection value of *Betaproteobacteria* and *Gammaproteobacteria* MAGs had increased. *Clostridia, Bacteroidia, Bacilli, Mollicutes, Negativicutes* MAGs had shown more contradictory behavior. It is worth noting that “survivor” bacteria MAGs, which were detected for one of the patients include *Bacteroides ovatus, stercoris, dorei, uniformis, thetaiotaomicron, fragilis, eggerthii*, and *vulgatus* as well as *Enterococcus faecium* and *faecalis*. Fold changes values by bacterial classes are presented in Figure S3.

HP eradication therapy affects not only bacteria but also archaea. We have obtained the archaeal MAG *Methanobrevibacter smithii* based on the patient's HP003 metagenomes. Detection value of this archaea decreased after HP eradication therapy. It is also worth noting that some microbial MAGs (including one archeal MAG) restored the detection value after therapy, which indicates the existence of compensatory processes leading to the restoration of richness and functions of the gut microflora. Full MAGs assembly statistic and detection value dynamics are shown in Table S7.

### 3.5. Links Between Metagenome-Assembled Genomes and Antibiotic Resistance Genes

It is logical to assume, that the bacteria that increased in relative abundance after HP eradication therapy harbor (or have acquired) features (such as ARGs) allowing them to protect themselves from antibiotics. Unfortunately, the contigs present in several genomes (for example, mobile elements carrying ARGs) may not be included in the corresponding MAGs during binning which makes the analysis difficult. In order to solve this problem, connections of the de Bruijn graphs can be used to restore the lost genes diversity of MAGs. For the first time, this approach was described in Barnum et al. (2018). We have modified the analysis protocol by replacing the complete metagenomic assembly with a local assembly using MetaCherchant algorithm. Also, there are new additional methods to restore the hidden gene diversity of MAGs (Brown et al., 2019).

**Figure 5:**
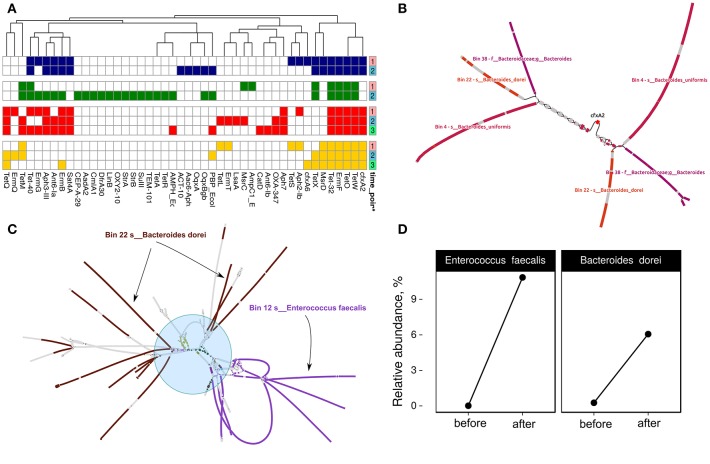
Discovery of links between MAGs and antibiotic resistance genes. **(A)** Resistome profiling of four patients gut metagenomes. Color shows individual patients (red—HP003, blue—HP009, green—HP010, yellow—HP028). Time points are denoted by a right color bar. **(B)** *CFX*-group gene within transposon-like structure is associated with three MAGs: Bin 38—unknown *Bacteroides*, Bin 22—*Bacteroides dorei*, and Bin 4—*Bacteroides uniformis*. Color shows areas of the MAGs close to the graph sequences according to the BLAST results (patient HP003, time points 2 and 3). **(C)** Multiple sets of ARGs such as *ermB, ermT, Ant6-Ia, Aph-III, Aph7, Sat4A, catA, tetL, tetM*, and *tetQ* (shown by the blue circle and different colors) are located close to Bin 12—*Enterococcus faecalis* and Bin 22—*B. dorei* MAGs (patient HP003, time point 2). **(D)** Relative abundance of *E. faecalis* and *B. dorei* in HP003 patient gut metagenomes before and after the HP eradication therapy according to MetaPhlan2 analysis.

MetaCherchant allows reconstruction of ARGs metagenomic context specified size, which speeds up the analysis. First, we performed resistome profiling by GROOT (graphing Resistance Out Of meTagenomes). This method is faster and more accurate compared to the ones used previously (Rowe and Winn, 2018). Determined resistome profiles are presented in Figure 5A. Secondly, the metagenomic context for each detected gene was assembled using MetaCherchant. Thirdly, the search of MAG sequences in the obtained contexts was performed using Bandage and BLAST. An example of how it works is shown in Figure 5B. This case was described in the MetaCherchant article earlier (Olekhnovich et al., 2017). The CFX group gene is located in the transposon, which in turn is located in three bacterial genomes (belonging to *Bacteroidales* according to BLAST and Kraken Wood and Salzberg, 2014 annotation). We have detected clear links between ARGs and MAGs in cases of (1) *isaA* gene and Bin 12—*E. faecalis* (patient HP003, time point 2); (2) *msrC* gene and Bin 36—*E. faecium* (patient HP003, time point 2) (3) *msrD* gene and Bin 11—*Streptococcus parasanguinis* (HP009 patient, time point 2), which shows that these ARGs are included in MAGs. Interestingly, the described MAGs increased in abundance after HP eradication therapy. The results are presented in Figure S4.

Some of the cases are complicated but they may be helpful in understanding the spread of ARGs in the human gut microbial communities. For example, Figure 5C indicates a hypothetical horizontal gene transfer between *B. dorei* and *E. faecalis*. Interestingly, this shared metagenomic context includes multiple co-localized ARGs such as *ermB, ermT, ant6-Ia, aph-III, aph7, sat4A, catA, tetL, tetM*, and *tetQ*. According to taxonomic profiling by MetaPhlAn2, we have seen an increase in relative abundance for *B. dorei* and *E. faecalis* in patient HP003's gut metagenome after HP eradication therapy (see Figure 5D). Thus, the co-localization of multiple ARGs in a bacterial organism could be a reason for their nonspecific increase.

### 3.6. Comparison of the Isolated *Enterococcus* spp. Genomes and MAGs

Since performing statistical analysis of 80 metagenomes revealed an increase in the relative abundance of *E. faecium* after HP eradication therapy (see Figure S2), enterococcal strains from samples of four patients were isolated (a total of 16 *E. faecium* and 3 *E. faecalis* strains) and sequenced using the Illumina HiSeq 2500 sequencing platform. We were able to recover the *E. faecium* strains from each patient before and after therapy except for the HP028 (only after). Two *E. faecium* MAGs (from HP003 and HP010 patients samples) were added to the comparative genomic analysis, too.

**Figure 6:**
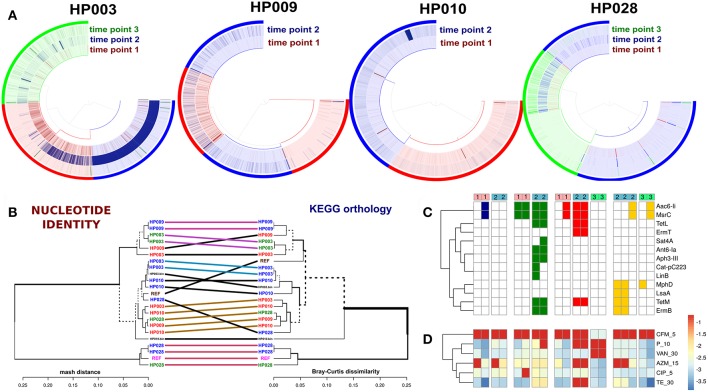
Comparative genomic analysis of *Enterococcus* spp. isolates. Genomes of *E. faecium* DO and *E. faecalis* V583 from NCBI were used as references. **(A)** Detection value of *Enterococcus* spp. isolates across related metagenomic samples. Isolates from different time points are shown by different colors (red—first point, blue—second point, green—third point). Every arc corresponds to one patient (captions under each plot). **(B)** Comparison dendrogram (average nucleotide identity, nucleotide identity and KEGG orthology groups distributions). Isolates from the 1st time point are colored red, isolates of 2nd and 3rd-time points are blue and green. The enterococci MAGs of HP003 and HP010 patients correspond to HP003.bin and HP010.bin signatures. **(C)** Resistome profiling results by GROOT (absence/presence matrix). Cell colors denoted patients' IDs (red—HP003, blue—HP009, green—HP010, yellow—HP028). Time points shown as top color bars. **(D)** The *in vitro* antibiotic susceptibility testing results demonstrate an increase in resistance, which is indicated by the transition from blue color to red.

The direct comparison of enterococci genomes with correspondent metagenomes revealed that in general, the detection values of studied isolates were statistically significantly increased after HP eradication therapy (Wilcoxon signed rank test, *p* < 0.01). Results are presented in Figure 6A. Enterococci genomes (included references and MAGs) were compared using two independent approaches. For compared genomes by nucleotide identity, we used Mash. Also, the distributions of KEGG orthology groups (KOs) were used for an additional collation method. The comparison dendrogram is presented in Figure 6B. Both methods agree that enterococci strains recovered from a certain patient at different time points were different. In the case of the HP003 patient, we have observed a high similarity between the genomes of isolated *E. faecium* and MAG. However, it is not true in the case of the HP010 patient. This may have resulted from the assembly of interstrain chimeric contigs. After HP eradication therapy, the ARGs numbers in enterococci genomes increased (Fisher exact test, *p* < 0.01). Additionally, we have seen that isolates decreased in susceptibility to azithromycin, penicillin, and tetracycline (Wilcoxon signed rank test with Benjamini-Hochberg correction for multiple testing, adj. *p* < 0.1). However, we have not observed a strong relationship between the resistome of a certain strain, the *in vitro* antibiotic susceptibility testing results (Figures 6C,D, respectively), and the detection value in the metagenomic sample collected after HP eradication therapy. The only HP003 isolates carrying 23S rRNA methyltransferase genes showed the complete resistance to azithromycin and the dramatic increase of their detection values. The HP009 isolates were completely resistant to azithromycin and carried no 23S rRNA methyltransferases genes. The HP028 isolates being completely resistant to azithromycin and carrying 23S rRNA methyltransferases genes did not dramatically increase their detection value.

## 4. Discussion

Changes in the microbial gut community structure caused by HP eradication therapy have already drawn attention of the scientific community. At the moment, we have found six publications describing the investigation of human intestinal microbiota under HP eradication therapy using metagenomic approaches. Five of them were performed by 16S rRNA gene sequencing (16S seq) method (Jakobsson et al., 2010; Yap et al., 2016; Hsu et al., 2018, 2019; He et al., 2019) and one was carried out using the whole-genome sequencing (WGS) protocol (Oh et al., 2016). So, enough 16S seq data has been accumulated to estimate the effects of HP eradication therapy on the taxonomic composition of gut microbiota, while further detailed analysis requires additional WGS data.

Here we present the WGS metagenomic study of the influence of HP eradication therapy to the human gut microbiota. We analyzed 80 metagenomic samples from 40 patients using the ABI/SOLiD 5500W sequencing platform. The general statistical analysis of taxonomic profiles showed a significant decrease in the alpha-diversity (reflects the microbiota richness) and in the relative abundance of *B. adolescentis* caused by therapy. An increase in relative abundance of *E. faecium* was also detected. Similar effects have been described earlier in two publications (Jakobsson et al., 2010; Hsu et al., 2018, 2019; He et al., 2019).

The model of gut microbiota ecological succession due to HP eradication therapy (see Figure 3B) was built using the balance dendrogram (CoDa dendrogram) approach (Egozcue and Pawlowsky-Glahn, 2005; Pawlowsky-Glahn and Egozcue, 2011). This analysis allows for consideration of co-links of taxa, such connections form the “balance.” It is suitable a decrease in the relative abundance of *Bifidobacteriaceae* and *Coriobacteriaceae*, which is associated with human colonic health and remission of inflammatory bowel diseases (IBD) (Morgan et al., 2012; Rooks et al., 2014; Maukonen et al., 2015). We have also seen a decreased relative abundance of short-chain fatty acid (SCFA) producing bacteria such as Eubacteriaceae, *Lachnospiraceae*, and *Ruminococcaceae* that is associated with a broad spectrum of disorders: Crohn's disease and other IBD (Nagao-Kitamoto and Kamada, 2017). Moreover, the increase in relative abundance of *Enterobacteriaceae* could be associated with many pathologies (Qin et al., 2012; Tong et al., 2013; Gevers et al., 2014; Matsuoka and Kanai, 2015; Chen and Devaraj, 2018; Tyakht et al., 2018). Also, the relative abundance of *Siphoviridae* bacteriophages was increased in relative abundance after HP eradication. The mentioned observations may be helpful in understanding the development of side effects of HP eradication therapy.

We have also discovered statistically significant differences in the functional potential of gut microbiota before and after HP eradication therapy. Anyway, seven KEGG pathways associated with cellular processes (ko02040: Flagellar assembly; ko02024: Quorum sensing; ko02030: Bacterial chemotaxis), genetic information processing (ko00970: Aminoacyl-tRNA biosynthesis; ko03010: Ribosome) and environmental information processing (ko02010: ABC transporters; ko02020: Two-component system) were underrepresented in the gene set analysis after HP eradication therapy. Five KEGG metabolic pathways associated with the metabolism of cofactors and vitamins (ko00130: Ubiquinone and other terpenoid-quinone biosynthesis; ko00785: Lipoic acid metabolism) and glycan biosynthesis and metabolism (ko00511: Other glycan degradation; ko00540: Lipopolysaccharide biosynthesis) were overrepresented.

The ABI/SOLiD sequencing technology performs very well if the data analysis is based on mapping the reads to reference genes/genomes. However, it guarantees a relatively low rate of sequencing errors for reads of maximum 50 bp length only. This reduces the possibility of generating a reliable *de novo* assembly for shotgun metagenomics (Oulas et al., 2015). For extended metagenomic analysis, ten samples from four patients have been re-sequenced using the Illumina HiSeq 2500 platform, which allowed for obtaining higher quality results. Illumina technology is considered the gold standard for metagenomic research, so many approaches for data analysis have been developed. For example, the genome-resolved metagenomics (GRM) technique allows assembling genomes from metagenomic data namely MAGs (metagenome-assembled genomes). It can be useful for identification of “hidden” trends, which cannot be identified via common taxonomic analysis. This approach unveils the so-called microbial “dark matter”—bacteria with unknown taxonomy which might constitute a significant part of the microbiota. It is also suitable for strain tracking, identification of “hidden” alterations, and other applications. Using the GRM approaches in our study, we confirmed and supplemented the known results concerning taxonomic reshaping. We observed almost complete reshaping of microbiota structure caused by HP eradication therapy. The majority of *Actinobacteria, Verrucomicrobia*, and *Synergistia* MAGs have decreased, while the *Betaproteobacteria* and *Gammaproteobacteria* MAGs increased. The other identified bacterial classes revealed more chaotic behavior. It is worth noting that some MAGs of *Bacteroides* (among them *ovatus, stercoris, dorei, uniforms, thetaiotaomicron, fragilis, eggerthii*, and *vulgatus*) and enterococci (*E. faecium* and *faecalis*) increased after the HP eradication therapy. Decrease in *Actinobacteria* and *Verrucomicrobia* and increase of *Bacteroidetes* and *Proteobacteria* corresponded to previously published data (Jakobsson et al., 2010; Hsu et al., 2018). However, we found an increased detection value in some *Bacteroides* MAGs and generally chaotic dynamics among *Bacteroidia* MAGs. These conflicting observations may be related to the characteristics of the sequencing and data analysis approaches, general patients conditions, diet, or any other significant factors and require additional research.

We have observed that the relative abundance of tetracycline resistance genes decreased after HP eradication therapy (*tetO, tetW*), while the relative abundance of ARGs to macrolide, beta-lactam and other tetracycline resistance genes increased (*ermB, CFX* group, and *tetQ*, respectively). It seems that the strains carrying ARGs, i.e., 23S rRNA methyltransferases, have gained an advantage under the strong selective pressure of therapy. Contradictory results on tetracycline resistance determinants are interesting. Here tetracycline was not included in the eradication scheme, and the acquisition of tetracycline resistance determinants does not give a significant advantage to their carriers under given circumstances. However, *tetQ, CFX* group, and *ermB* genes might be co-localized in one mobile element and/or bacterial genome. This hypothesis was confirmed by the local assembly of the *tetQ* gene metagenomic context using MetaCherchant (the local metagenomic assembly across target sequence). The *tetQ* gene was co-localized with multiple ARGs included in *E. faecalis* and *B. dorei* genomes (*B. dorei* also carrying *CFX*). Similar effects may be indicated by increasing the numbers of detected ARGs in the metagenomes after HP eradication. This case also may be the result of an HGT event between distant taxa. Enterococci can accommodate different mobile elements and spread antibiotic resistance factors to a broad spectrum of Gram-positive and Gram-negative bacteria. Therefore, they are of interest to antibiotic resistance researchers. For example, the transfer of vancomycin resistance to strains of *Staphylococcus aureus* was reported (Lebreton et al., 2017).

Relative abundance of *E. faecium* has increased according to metagenomic analysis. This effect was confirmed in a study on *Enterococcus* spp. isolates, which were obtained from stool samples. Isolates showed a statistically significant increase in detection value, the resistance to azithromycin, tetracycline, and penicillin *in vitro* and numbers of ARGs after therapy. However, not all cases of significant increase are associated with *in vitro* resistance to macrolides and/or 23S rRNA methyltransferases presence in the genome. Thus, the mechanism of *Enterococcus* increase is still not clear and requires additional research.

However, the obtained results can be specific for the antibiotics applied in the HP eradication. The specific pool of ARGs for which we observed quantitative alterations obviously reflects the set of antibiotics included in the Maastricht protocol. They are likely to change if other classes of antibiotics are applied for treating HP or other diseases. However, the general effects we observe—depletion of beneficial taxa and rise of opportunists enriched in ARGs—will likely remain.

## 5. Conclusions

HP eradication causes multiple shifts and alterations in the intestinal microbiota and leads to a specific accumulation of macrolide resistance genes. The microbial community has changed toward the reduction of overall metabolic potential and the increase of potential survival mechanisms. Surprisingly, with such a hard antibacterial pressure, the community is restructured, many bacteria can survive, and there is a probability of the emergence of bacteria with multiple antibiotic resistance. Moreover, many studies of HP eradication therapy's influence on human gut microbiota have noted a decrease in *Bifidobacterium* spp. as well as associated taxonomic levels. *Bifidobacterium* is involved in carbohydrate metabolism, prevents the colonization of gut mucosa by opportunistic bacterial species (Hütt et al., 2006; Martins et al., 2010), and produces other beneficial effects for the host. It suggests that including probiotic *Bifidobacterium* in the treatment regimen might reduce the side effects of HP eradication. Understanding of the mechanisms involved in gut microbiome dynamics following antibiotic treatment will help to reduce the potential health hazards linked to drug resistance including antibiotic-associated diarrhea, *Clostridium difficile* infection, and post-FMT invasion of pathogenic resistant species.

## Data Availability

Raw metagenomic reads for 80 fecal samples from 40 patients sequenced by ABI/SOLiD 5500W are deposited in the NCBI Archive (project ID: PRJNA413659; Web-address: https://www.ncbi.nlm.nih.gov/bioproject/413659). Raw reads for 10 samples from 4 patients sequenced by Illumina HiSeq 2500 are deposited in the NCBI Archive (project ID: PRJEB18265; Web-address: https://www.ncbi.nlm.nih.gov/bioproject/?term=PRJEB18265). Additional supplementary materials such as MAGs fasta files, Anvi'o profiles, assembled genomes of *Enterococcus* spp., GROOT graphs, MetaCherchant graphs and others are available at http://download.ripcm.com/resistome_project.

## Ethics Statement

This study was approved by the ethical committee of the Kazan State University (Protocol No. 3, Apr 24, 2015). Before the start of the study, each patient signed an informed consent.

## Author Contributions

EK and TG conceived the study. VG, EI, EK, TG, and AM supervised the study. AP and AT coordinated the project. EK, TG, EB, MVM, VB, AL, SM, and MIM performed the experiments. EO, NP, BK, and AS performed data analysis. EO, AM, AT, ES, AP, AS, DK, and OG prepared the manuscript. SA, RA, and DS performed sample collection.

### Conflict of Interest Statement

The authors declare that the research was conducted in the absence of any commercial or financial relationships that could be construed as a potential conflict of interest.

## Abbreviations

CoDa: compositional data
KEGG: Kyoto Encyclopedia of Genes and Genomes
KO: KEGG orthology group
GSA: gene set analysis
OTU: operational taxonomic unit
SNV: single nucleotide variant
GRM: genome-resolved metagenomics
MAG: metagenome assembled-genome
ARG: antibiotic resistance gene
HGT: horizontal gene transfer
IBD: inflammatory bowel diseases
SCFA: short chain fatty acid
FMT: fecal mass transplantation.
