# Supplementary material for: Shifts in the Human Gut Microbiota Structure Caused by Quadruple Helicobacter pylori Eradication Therapy
Source: Front Microbiol. 2019 Aug 27;10:1902. doi: 10.3389/fmicb.2019.01902 (PMC6718723; doi:10.3389/fmicb.2019.01902)
Supplement: Supplementary file 2 [file Data_Sheet_1.pdf]

Supplementary Materials:
Shifts in the human gut microbiota structure caused by quadruple *Helicobacter pylori* eradication therapy

SUPPLEMENTARY FIGURES

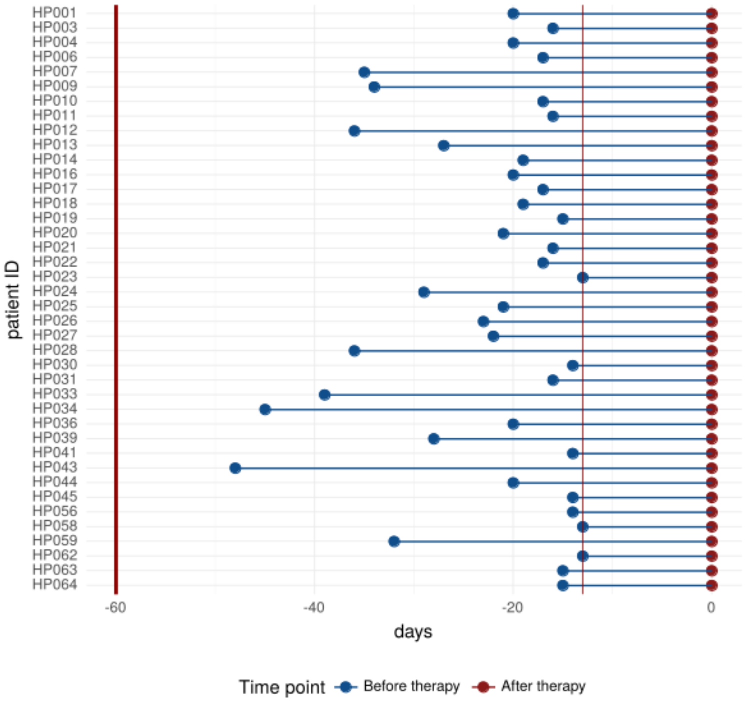

Figure S1. Sampling points schematic visualization. The vertical red line (X = - 14) demonstrates the beginning of taking medications.

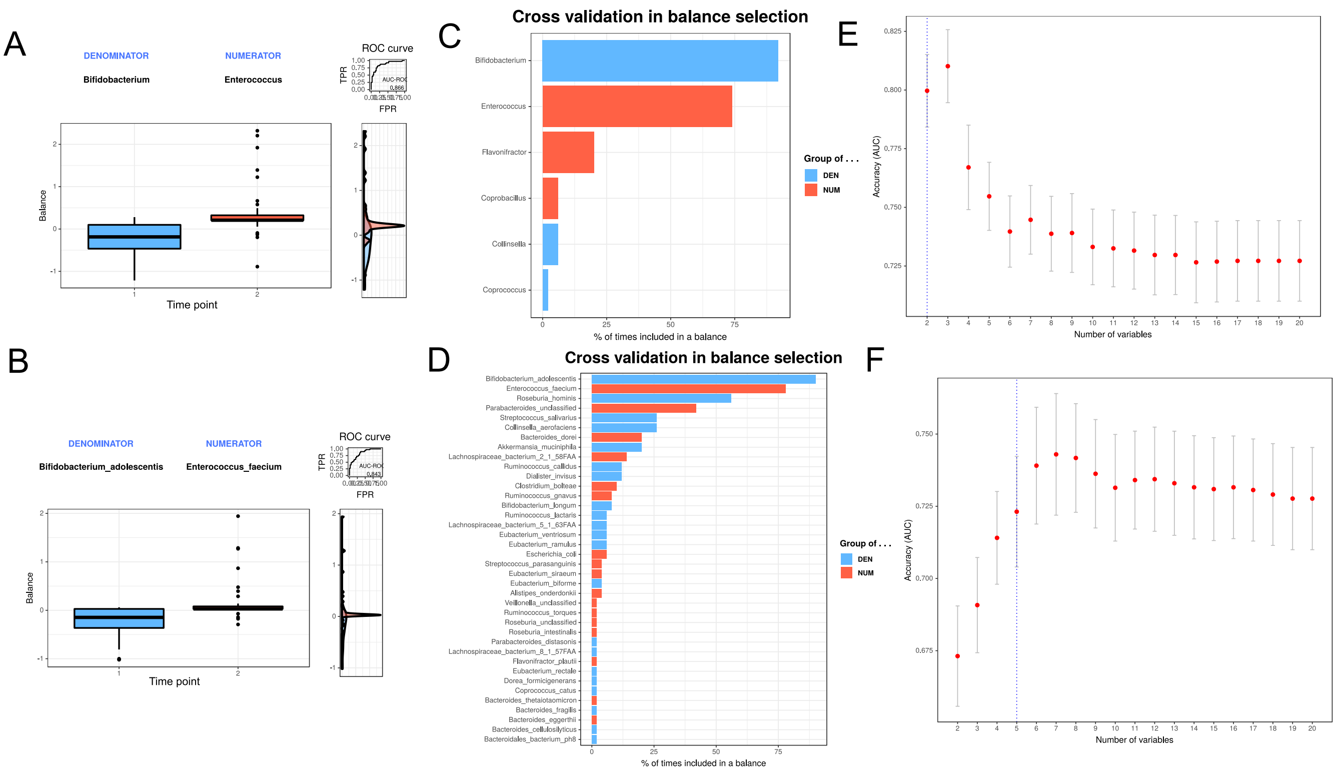

Figure S2. Selbal analysis results. (A-B) Description of the global balance for eradication therapy. The two groups of genera forming global balance are presented in the upper part of the plot. The boxplot shows the distribution of the balance scores for sample groups before and after eradication. The right side contains the ROC curve and the density plot. **(C-D)** Cross-validation in balance selection. Taxa are shown along the X-axis, fraction of times when a taxon was included in balance – along the Y-axis. **(E-F)** Mean area under the ROC curve (AUC) as a function of the number of components included in the balance in the cross-validation process. The optimal number of components according to the ”1st rule” is highlighted with a vertical dashed line. Analysis was performed according to Rivera-Pinto et al. (2018) [DOI: 10.1128/mSystems.00053-18].

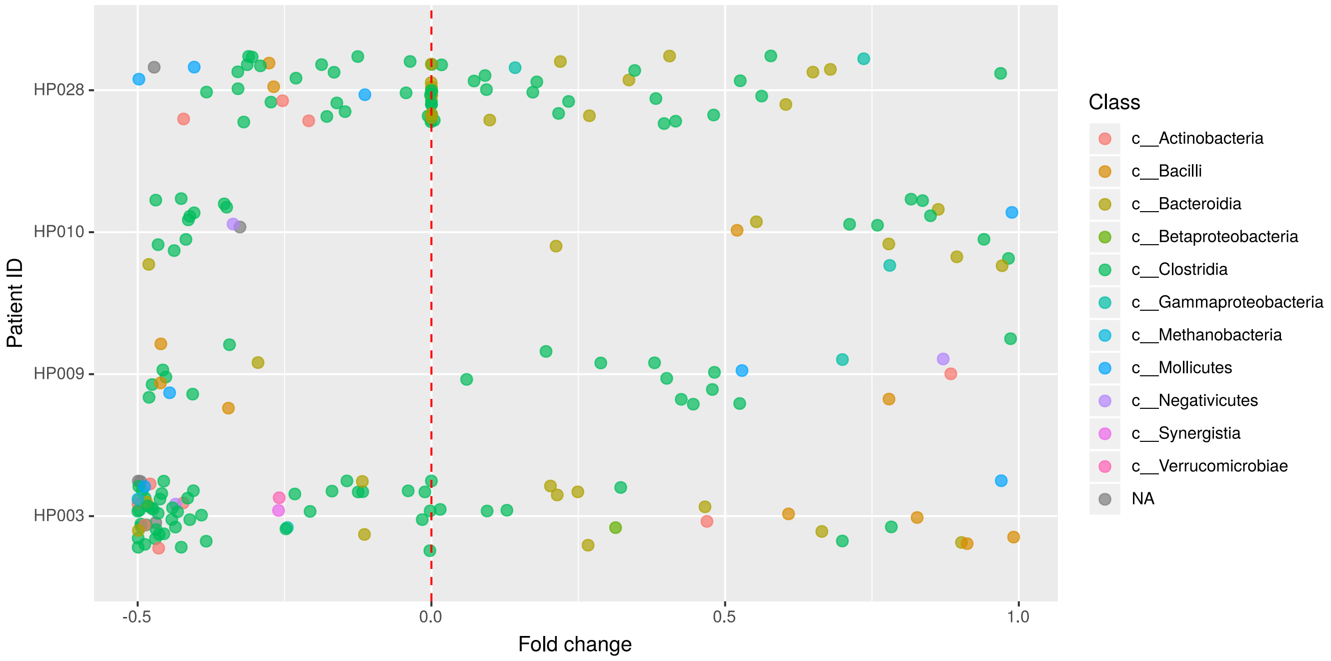

Figure S3. Fold changes of MAGs detection value between 1st and 2nd time points. The values > 0 (< 0) show MAGs increased (decreased) in abundance after HP eradication. The different colors denote different microbial classes.

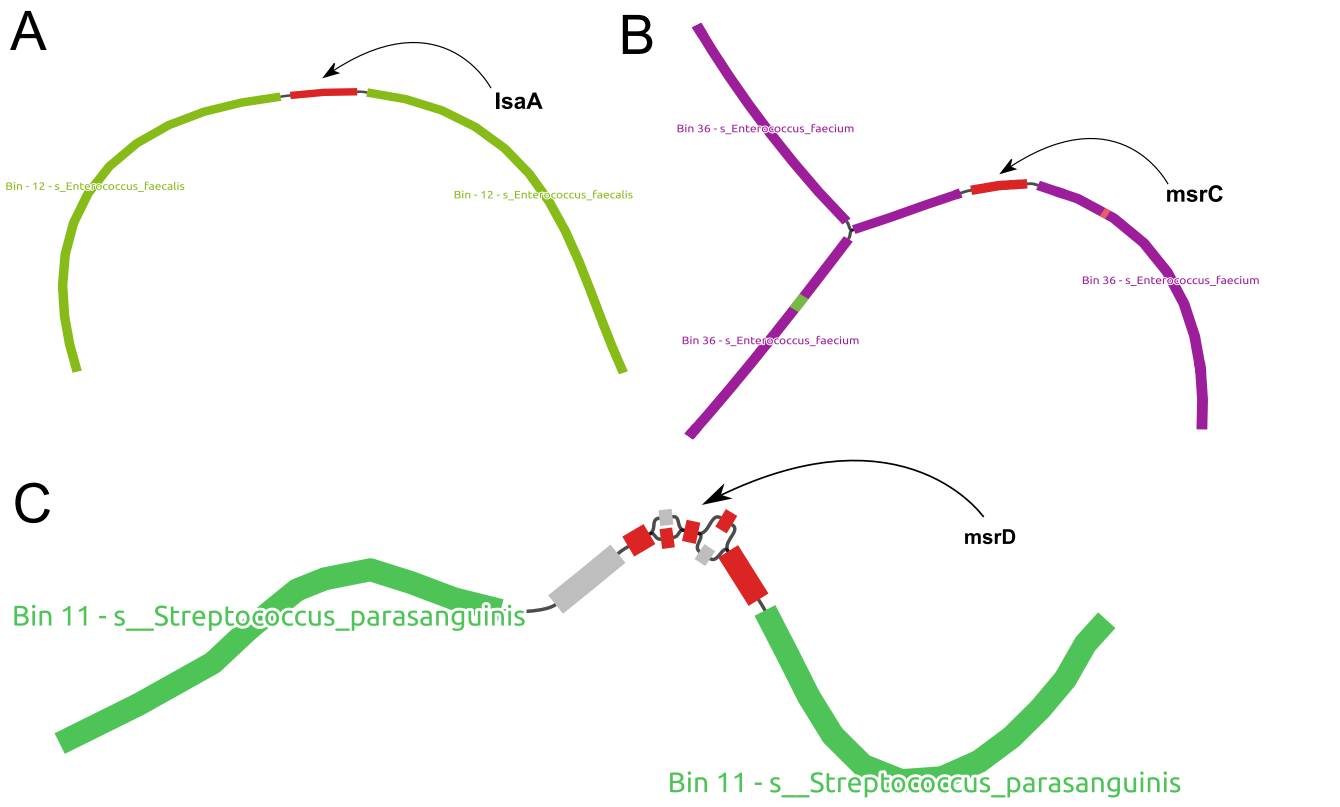

Figure S4. Examples of links between MAGs and ARGs for patient gut metagenomes. (A) *LsaA* gene and *Enterococcus faecalis* MAG (bin 12, HP003 patient, time point 2) **(B)** *MsrC* gene and *Enterococcus faecium* MAG (bin 36, HP003 patient, time point 2) **(C)** *MsrD* gene and *Streptococcus parasanguinis* MAG (bin 11, HP009 patient, time point 2).
